# Inverse Relationship between Metabolic Syndrome and 25-Hydroxyvitamin D Concentration in Elderly People without Vitamin D deficiency

**DOI:** 10.1038/s41598-018-35229-2

**Published:** 2018-11-19

**Authors:** Chun-Min Wang, Chin-Sung Chang, Yin-Fan Chang, Shin-Jiuan Wu, Ching-Ju Chiu, Meng-Tzu Hou, Chuan-Yu Chen, Ping-Yen Liu, Chih-Hsing Wu

**Affiliations:** 1 Department of Neurology, National Cheng Kung University Hospital and College of Medicine, National Cheng Kung University, Tainan, Taiwan; 2 Department of Family Medicine, National Cheng Kung University Hospital and College of Medicine, National Cheng Kung University, Tainan, Taiwan; 3 Department of Food Nutrition, Chung Hwa University Medical Technology, Tainan, Taiwan; 4 Institute of Gerontology, College of Medicine, National Cheng Kung University, Tainan, Taiwan; 5 Department of Physical Therapy and Assistive Technology, National Yang-Ming University, Taipei, Taiwan; 6 Department of Internal Medicine, Division of Cardiology, National Cheng Kung University Hospital and College of Medicine, National Cheng Kung University, Tainan, Taiwan

## Abstract

Vitamin D status is inversely associated with the prevalence of metabolic syndrome (MetS). Whether this is true in the elderly without vitamin D deficiency is rarely investigated. Our data source is a cross-sectional survey of 1,966 community-dwelling elderly Taiwanese in 2012. An overnight fasting blood were obtained for biochemistry variables. Vitamin D deficiency was defined as serum 25-hydroxyvitamin D3 [25(OH)D] concentration <20 ng/mL. MetS is defined using modified ATP-III criteria. Of 523 participants without vitamin D deficiency (Men/Women = 269/254, age = 76.0 ± 6.2 years old [65–102 years old]), mean 25(OH)D was 44.0 ± 11.1 ng/mL, and the MetS prevalence of MS was 46.5%. Serum 25(OH)D was negatively associated with osteocalcin, the homeostatic model assessment insulin resistance (HOMA-IR) index, body mass index (BMI), and glycated hemoglobin A1c. Participants with more MetS features have lower serum 25(OH)D and osteocalcin. Binary logistic regression models showed that 25(OH)D, physical activity, and osteocalcin were negatively independent MetS factors, but that the HOMA-IR index, BMI, and being female were positively independent factors. The risk of MetS was progressively lower along with the increased 25(OH)D concentration, even above 60 ng/mL. In conclusion, a low 25(OH)D concentration is an independent risk factor for MetS in elderly people without vitamin D deficiency.

## Introduction

Metabolic syndrome (MetS) is a cluster of abnormal metabolic markers: hypertension, insulin resistance, proinflammatory processes, dyslipidemia, and abnormal fat distribution. It is considered a major risk factor for type 2 diabetes mellitus (DM), cardiovascular disease (CVD), and all-cause mortality^1,2^. Because its prevalence increases with age, preventing it is important in aging societies.

Advanced age is also a known risk factor for vitamin D deficiency^3,4^. Vitamin D has been a focus of attention in recent decades because it is involved in bone metabolism and chronic diseases like MetS, type 2 DM, CVD, cancer, and cognitive dysfunction^5^. Several mechanisms linking vitamin D deficiency and MetS have been proposed: the alteration of the differentiation of preadipocytes, the inhibition of pancreatic β cell function, insulin resistance (IR), systemic inflammation, and elevated blood pressure induced by activating the renin-angiotensin-aldosterone system (RAAS)^6^. Several biomarkers have been evaluated to explain these mechanisms. The homeostatic model assessment insulin resistance (HOMA-IR) index is useful for estimating pancreatic β cell function and IR, and many studies have reported a negative association between 25(OH)D concentration and the HOMA-IR index^7^. However, one study^8^ of elderly Chinese found no association between IR and vitamin D status in patient with type 2 DM. High sensitivity C-reactive protein (hsCRP) is a commonly used biomarker for systemic inflammation, and several studies^9^ found an inverse relationship between vitamin D status and inflammation markers.

In addition to its role in bone mineralization and calcium ion homeostasis, osteocalcin induces pancreatic β cells to release more insulin and adipocytes to release adiponectin, which increases one’s sensitivity to insulin^10^. In the past decade, osteocalcin has been reported^11^ to be inversely associated with body adiposity and MetS, but it might predict MetS in the elderly.

Although most studies focus on the differences of metabolic aberrations between people with and without vitamin D deficiency, almost none focus on the elderly without vitamin D deficiency. The interrelationships among vitamin D, osteocalcin, the HOMA-IR index, hsCRP, and MetS are complicated and inadequately evaluated. Thus, we investigated these associations in the elderly without vitamin D deficiency, defined as 25-hydroxyvitamin D [25(OH)D] <20 ng/mL^11^. We hypothesized that there would be a consistently inverse association between 25(OH)D and MetS, even in the elderly without vitamin D deficiency.

## Results

After excluding participants with vitamin D deficiency, we reviewed and analyzed 523 medical records (269 men, 254 women; mean age: 76.0 ± 6.2 years old; age range: 65–102 years) with complete data. The average 25(OH)D concentration was 44.0 ± 11.1 ng/mL (range: 20–70 ng/mL), and the prevalence of MetS was 46.5%. The Mini Nutrition Assessment (MNA) showed that 82.2% of the participants were well-nourished, and the Short Portable Mental Status Questionnaire (SPMSQ) showed that 89% had no or mild cognitive impairment. Participants with MetS were more often female, less literate, and had higher BMI, higher high sensitivity C-reactive protein (hsCRP), lower 25(OH)D, and lower osteocalcin levels (Table 1). Serum 25(OH)D concentrations were not significantly different between participants taking (40.1 ± 11.7; n = 5) and not taking (43.9 ± 11.2; n = 518) vitamin D supplements (Appendix 1). Vitamin D concentrations were more often relatively lower (20 ng/mL ≤ 25(OH)D < 30 ng/mL) in women, those living with a partner, with greater cognitive impairment, who smoked less, and who had a higher fasting glucose level, homeostasis model assessment insulin resistance (HOMA-IR) index, and osteocalcin level (Appendix 2). Similar findings were presented for lower 25(OH)D subjects defining as 20 ng/mL ≤ 25(OH)D < 40 ng/mL (Appendix 3).

**Table 1:** Demographic and laboratory data of MetS^−^ and MetS^+^ elderly.

Variable	All	MetS^−^	MetS^+^	p value
**Number**	523	280	243	
**Age** (**years**)*	76 ± 6.2	76.8 ± 6.3	75.1 ± 6.1	0.002
**Male**	269 (51.4%)	172 (61.4%)	97 (39.9%)	<0.001
**Employed**	230 (44.1%)	130 (46.4%)	100 (41.3%)	0.241
**Lived with partner**	436 (83.4%)	236 (84.3%)	200 (82.3%)	0.544
**Literate**	269 (51.0%)	158 (56.2%)	111 (45.1%)	0.011
**Habitual alcohol drinking**	84 (16.1%)	49 (17.5%)	35 (14.4%)	0.336
**Smoking**				0.226
PY = 0	389 (74.4%)	200 (71.4%)	189 (77.8%)	
0–30 PY	44 (8.4%)	25 (9%)	19 (7.8%)	
≥30 PY	90 (17.2%)	55 (19.6%)	35 (14.4%)	
**Physical activity** (**IPAQ-short form**)				0.209
Low	172 (32.9%)	83 (29.7%)	89 (36.6%)	
Middle	171 (32.7%)	93 (33.3%)	78 (32.1%)	
High	180 (34.4%)	104 (37.1%)	76 (31.3%)	
**Body mass index** (**kg/m**^**2**^)	24.5 ± 3.8	22.9 ± 3.3	26.3 ± 3.5	<0.001
**Waist circumference** (**cm**)	87.2 ± 10.1	82.9 ± 9.3	92.1 ± 8.7	<0.001
**Components of metabolic syndrome**				
Central obesity	319 (61.0%)	101 (36.1%)	218 (89.7%)	<0.001
Hypertension	391 (74.8%)	171 (61.1%)	220 (90.5%)	<0.001
High fasting glucose	228 (43.6%)	70 (25.0%)	158 (65.0%)	<0.001
Hypertriglyceridemia	127 (24.3%)	14 (5.0%)	113 (46.5%)	<0.001
Low HDLC	186 (35.7%)	23 (8.2%)	163 (67.1%)	<0.001
**Mini-nutritional assessment**				0.719
Malnourished (<17.0)	3 (0.6%)	2 (0.7%)	1 (0.4%)	
At risk of malnutrition (17–24)	90 (17.2%)	51 (18.2%)	39 (16.1%)	
Well-nourished (≥24.0)	430 (82.2%)	227 (81.1%)	203 (83.5%)	
**SPMSQ**				0.899
No or mild cognitive impairment	468 (89.5%)	251 (89.6%)	217 (89.3%)	
Moderate to severe cognitive impairment	55 (10.5%)	29 (10.4%)	26 (10.7%)	
**25**(**OH**)**D** (**ng/mL**)	44.0 ± 11.1	45.9 ± 11.4	41.7 ± 10.3	<0.001
**hsCRP > 3** (**mg/L**)	103 (19.7%)	45 (16.1%)	58 (23.9%)	0.025
**Log** (**HOMA-IR**)	0.23 ± 0.34	0.086 ± 0.28	0.402 ± 0.33	<0.001
**Log** (**osteocalcin**) (**ng/dL**)	1.29 ± 0.21	1.31 ± 0.20	1.26 ± 0.21	0.011

Data expressed as number (percent) or mean ± standard deviation. Continuous data were analyzed using independent-sample *t* tests; dichotomous data were analyzed using χ^2^ tests. *Using Mann-Whitney tests due to non-gaussian distribution. PY: pack-year; HDLC: high-density lipoprotein cholesterol; HOMA-IR: homeostatic model assessment insulin resistance; hsCRP: high-sensitivity C-reactive protein; IPAQ: International Physical Activity Questionnaire; SPMSQ: Short Portable Mental Status Questionnaire.

**Figure 1:**
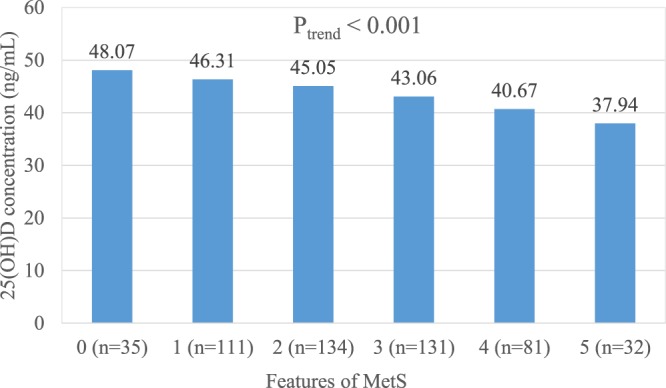
Mean serum 25(OH)D concentration (ng/mL) in 523 elderly without vitamin D deficiency but with 0 to 5 features of the metabolic syndrome. (P_trend_ < 0.001).

**Figure 2:**
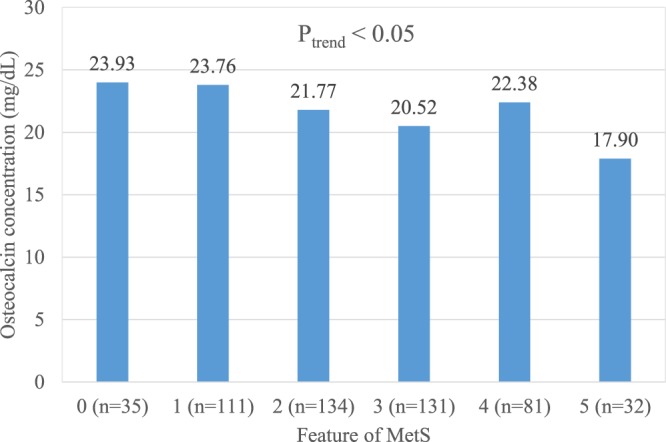
Mean osteocalcin (ng/dL) concentration in 523 elderly without vitamin D deficiency but with 0 to 5 features of the metabolic syndrome (P_trend_ < 0.05).

**Figure 3:**
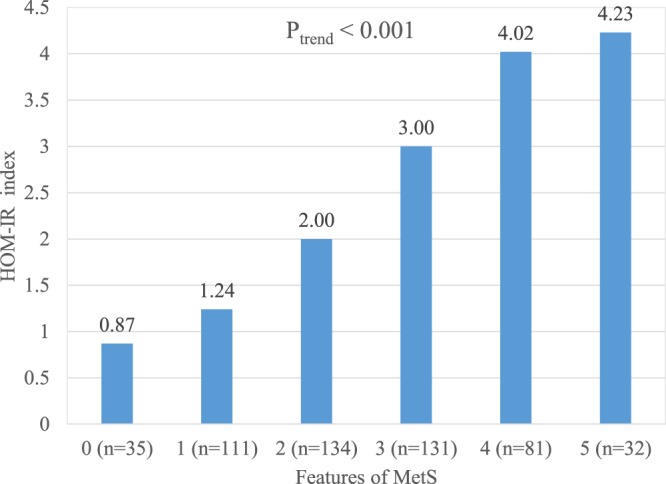
Mean HOMA-IR index in 523 elderly without vitamin D deficiency but with 0 to 5 features of the metabolic syndrome (P_trend_ < 0.001).

**Figure 4:**
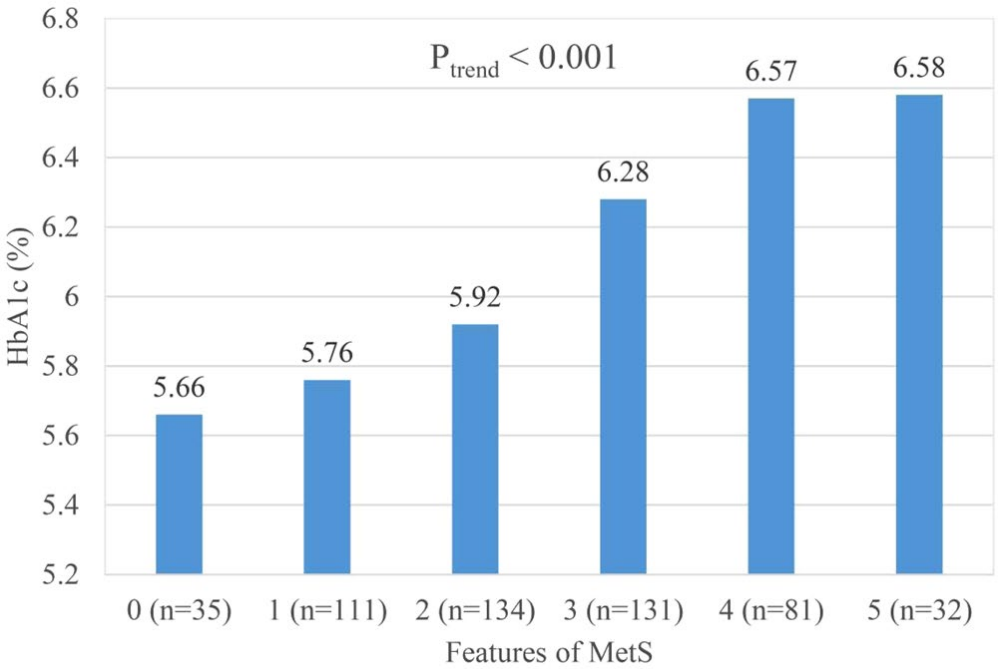
Mean glycated hemoglobin A1c (HbA1c) in 523 elderly without vitamin D deficiency but with 0 to 5 features of the metabolic syndrome (P_trend_ < 0.001).

Serum 25(OH)D was significantly negatively associated with osteocalcin (r = 0.098, p < 0.05), the HOMA-IR index (r = −0.134, p < 0.01), intact parathyroid hormone (iPTH) (r = −0.156, p < 0.01), and body mass index (BMI) (r = −0.107, p < 0.05) (Table 2). Participants with more features of MetS tended to have relatively lower serum 25(OH)D (p_trend_ < 0.001) and osteocalcin levels (p_trend_ < 0.05); and a higher HOMA-IR index (p_trend_ < 0.001) and HbA1c levels (p_trend_ < 0.001) (Figs 1–4).

**Table 2:** Pearson correlation coefficients between 25(OH)D and related metabolic variables in 523 elderly without vitamin D deficiency (25(OH)D ≥ 20 ng/mL)

Variable	25(OH)D	iPTH	hsCRP	Osteocalcin^#^	HOMA-IR	BMI	WC	HbA1c
	(ng/mL)	(pg/mL)	(mg/L)	(ng/mL)	^#^	(kg/m^2^)	(cm)	(%)
25(OH)D	1	—	—	—	—	—	—	—
iPTH	−0.156**	1	—	—	—	—	—	—
HsCRP	0.032	0.024	1	—	—	—	—	—
Osteocalcin^#^	−0.098*	0.404**	0.014	1	—	—	—	—
HOMA-IR^#^	−0.134**	0.005	0.078	–0.123**	1	—	—	—
BMI	−0.107*	0.029	0.086*	–0.135**	0.505**	1	—	—
WC	−0.030	0.019	0.139**	–0.177**	0.493**	0.87**	1	—
HbA1c	−0.087*	−0.46	0.029	–0.219**	0.411**	0.183**	0.222*	1

iPTH: intact parathyroid hormone, hsCRP: high-sensitivity C-reactive protein, BMI: body mass index, WC: waist circumference, HbA1c: hemoglobin A1c, HOMA-IR: homeostatic model assessment insulin resistance, ^#^log transformation, *p < 0.05, **p < 0.01.

We used four logistic regression models to analyze the odds ratio (OR) of MetS. Model 1 showed that age (OR = 0.95, 95% CI: 0.92–0.98, p = 0.002), physical activity (OR = 0.57, 95% CI: 0.35–0.93, p = 0.026), serum 25(OH)D (OR = 0.98, 95% CI: 0.96–0.99, p = 0.007), and osteocalcin level (OR = 0.18, 95% CI: 0.07–0.46, p < 0.001) were negatively independent factors, but that being female (OR = 2.8, 95% CI: 1.59–4.92, p < 0.001) was a positive independent factor. In Models 2 and 3, the HOMA-IR index and BMI, respectively, were added for analysis. The significance of vitamin D and osteocalcin was lower but still significant. In Model 4, serum 25(OH)D was a significant negative independent factor (OR = 0.98, 95% CI: 0.96–0.99, p = 0.047), as were BMI and the adjusted HOMA-IR index (Table 3).

**Table 3:** Binary logistic regression model for factors significantly associated with the presence of MetS in 523 elderly without vitamin D deficiency (25(OH)D ≥ 20 ng/mL).

Nagelkerke R^2^		Model 1	Model 2	Model 3	Model 4
		0.16	0.36	0.35	0.43
Variable	Category	OR (95% CI)	OR (95% CI)	OR (95% CI)	OR (95% CI)
Age (years)		0.95 (0.92–0.98)***	0.98 (0.94–1.02)	0.98 (0.94–1.02)	0.99 (0.95–1.03)
Sex	Female	2.80 (1.59–4.92)***	2.28 (1.24–4.21)**	2.67 (1.44–4.96)**	2.27 (1.20–4.29)*
BMI (kg/m^2^)		—	—	1.37 (1.27–1.47)***	1.26 (1.16–1.36)***
Live alone	Yes	1.32(0.79–2.19)	1.32 (0.76–2.31)	1.28 (0.74–2.23)	1.30 (0.73–2.33)
Employed	Yes	1.12 (0.72–1.73)	0.80 (0.49–1.31)	0.96 (0.59–1.56)	0.80 (0.47–1.33)
Literate	Yes	0.94 (0.60–1.48)	0.74 (0.45–1.22)	0.87 (0.53–1.43)	0.75 (0.44–1.27)
Smoking (PYs)	PYs = 0 (reference)	1	1	1	1
	0–30 PYs	1.51 (0.71–3.21)	1.20 (0.52–2.76)	1.70 (0.74–3.92)	1.37 (0.57–3.29)
	≥30 PYs	1.50 (0.80–2.80)	1.22 (0.60–2.48)	1.46 (0.74–2.89	1.23 (0.59–2.55)
Alcohol drinking	Yes	1.23 (0.67–2.24)	1.57 (0.80–3.11)	1.31 (0.68–2.53)	1.55 (0.77–3.13)
Physical activity	Low (reference)	1	1	1	1
	Middle	0.57 (0.35–0.94)*	0.50 (0.29–0.87)*	0.59 (0.34–1.01)	0.52 (0.29–0.92)*
	High	0.59 (0.34–1.02)	0.46 (0.25–0.85)*	0.49 (0.27–0.91)*	0.42 (0.22–0.80)*
Malnutrition	No (reference)	1	1	1	1
	At risk	3.12 (0.26–37.58)	4.47 (0.18–109.5)	1.12 (0.09–13.58)	1.79 (0.10–32.60)
		3.61 (0.31–42.40)	5.12 (0.21–122.3)	0.82 (0.07–9.69)	1.49 (0.08–26.59)
Mental impairment	Moderate to severe	1.16 (0.61–2.19)	1.57 (0.77–3.21)	1.29 (0.63–2.63)	1.53 (0.73–3.20)
25(OH)D (ng/mL)		0.98 (0.96–0.99)**	0.98 (0.96–0.99)*	0.98 (0.96–0.99)*	0.98 (0.96–0.99)*
Log (HOMA-IR)		—	33.8 (14.9–76.8)***	—	13.24 (5.65–31.05)***
Log (osteocalcin) (ng/dL)		0.18 (0.07–0.46)***	0.29 (0.10–0.82)*	0.25 (0.09–0.70)**	0.34 (0.12–1.00)
hsCRP (mg/L)		1.03 (0.99–1.06)	1.00 (0.97–1.05)	1.01 (0.97–1.05)	1.00 (0.94–1.04)

OR: odds ratio; CI: confidence interval; PYs: packs per year; HOMA-IR: homeostatic model assessment insulin resistance; BMI: body mass index; hsCRP: high sensitivity C-reactive protein. *p < 0.05, **p < 0.01, ***p < 0.001.

**Figure 5:**
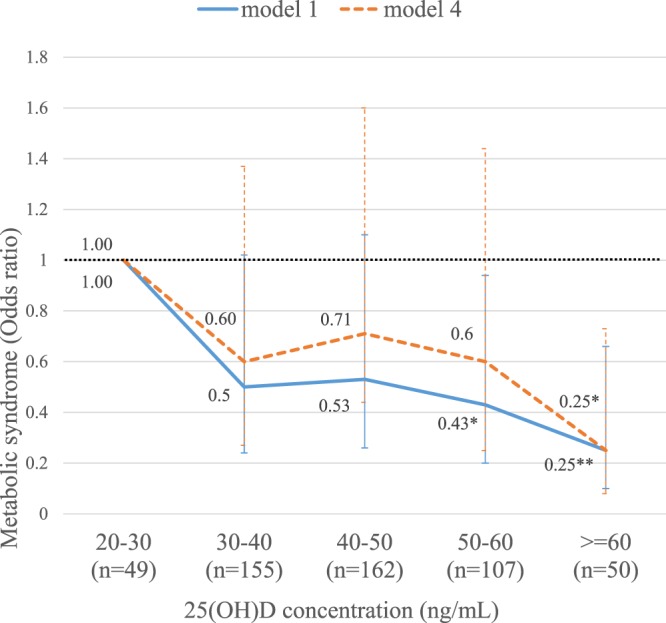
Risk of metabolic syndrome by 5 subgroups of 25(OH)D concentration. Data was derived from Appendix 6. Model 1 (—): adjusted with all major variables, except body mass index and HOMA-IR index, Model 4(---): adjusted with all variables, include body mass index and HOMA-IR, *p < 0.05; **p < 0.01.

Using the same binary logistic regression models to analyze participants with either vitamin D > 32 ng/mL^12^ or vitamin D > 40 ng/mL showed that 25(OH)D was an independent factor for MetS (Appendices 4 and 5). We further categorized 25(OH)D concentration into 5 subgroups [20 ng/mL ≤ 25(OH)D < 30 ng/mL (n = 49), 30 ng/mL ≤ 25(OH)D < 40 ng/mL (n = 155), 40 ng/mL ≤ 25(OH)D < 50 ng/mL (n = 162), 50 ng/mL ≤ 25(OH)D < 60 ng/mL (n = 107), 25(OH)D ≥ 60 ng/mL (n = 50)]. The odds ratio (OR) showed a decreasing trend along with the increased 25(OH)D concentration (Fig. 5). The ORs for metabolic syndrome reached significance at 50 ng/mL ≤ 25(OH)D < 60 ng/mL (OR = 0.43, 95% CI: 0.20–0.94, p = 0.03) and 25(OH)D ≥ 60 ng/mL (OR = 0.25, 95% CI: 0.10–0.66, P = 0.005) in Model 1 (Appendix 6), and at 25(OH)D ≥ 60 ng/mL (OR = 0.25, 95% CI: 0.08–0.73, p = 0.012) in Model 4 (Appendix 6).

## Discussion

Our findings that being female, a higher HOMA-IR index, lower physical activity, osteocalcin^13^, and vitamin D concentrations are independent risk factors of MetS are consistent with other studies^1,2,7,14^. Moreover, our study confirmed these relationships in elderly people without vitamin D deficiency or insufficiency.

Old age is a known risk factor for vitamin D deficiency^3^, but physical activity, exposure to sunlight, skin pigmentation, clothing, diet, and nutritional status might affect serum 25(OH)D concentrations^14,15^. Our participants lived in an area with abundant annual sunshine, and 44.3% of them were still active farmers. The MNA showed that they were robustly nourished, which might explain their relatively low prevalence of vitamin D deficiency or insufficiency and indicate a nature-nurture relationship between MetS and vitamin D.

The seasonal variation of serum 25(OH)D might be concerned but not consistent in different regions^14,16,17^. In a large Taiwan National Nutrition Survey^18^ and studies in Taiwan^19,20^, the seasonal variation of serum 25(OH)D was trivial (less than 3 ng/ml). As a randomized controlled trial found that vitamin D supplements were beneficial for lowering IR, especially when serum 25(OH)D concentration was ≥32 ng/mL (80 nmol/L)^12^. We used a similar binary logistic regression analysis in selected participants with serum concentrations of 25(OH)D ≥32 ng /mL or ≥40 ng/mL to reflect the possible effect of seasonal changes of vitamin D status. Vitamin D status was consistently an independent variable for MetS.

The core metabolic abnormality in MetS is IR. Other studies^21–23^ have reported that vitamin D modulates the effect of insulin by directly increasing the expression of insulin receptor, which increases insulin responsiveness for transporting glucose, or by indirectly regulating extracellular calcium in insulin-responsive skeletal muscle and adipose tissue. However, obesity might complicate the relationship between vitamin D and MetS. Obesity is associated with IR and with high blood pressure and dyslipidemia; thus, it is a significant risk factor for MetS^1^. There is a complex interrelationship between adiposity and vitamin D. Because vitamin D is fat-soluble, it can be sequestered, or diluted, in enlarged adipose compartment, thereby lowering serum 25(OH)D concentrations^24^. Most studies^25,26^ have reported that serum vitamin D status falls as adiposity increases. A large bidirectional genetic study^27^ hypothesized that a higher BMI leads to lower 25(OH)D, but that “any effects of lower 25(OH)D increasing BMI are likely to be small”. We found that vitamin D status negatively correlated with HOMA-IR and BMI. However, although we added both BMI and HOMA-IR to the analysis, vitamin D remained a significant independent negative factor for MetS. The regression models for patients with 25(OH)D concentrations ≥32 and ≥40 ng/mL were not significantly different (Appendices 4 and 5). This might indicate that there is an unidentified underlying mechanism other than increased IR and obesity that links vitamin D and MetS.

Other mechanisms linking vitamin D and MetS have been proposed^5^. The roles of vitamin D in immune system had been extensively studied in recent 10 years^28,29^. Vitamin D and its analogues inhibit the production of interleukin-2 and interferon-γ, and they stimulate the effects of T-helper type 2 lymphocytes, which leads to a reduction in matrix metalloproteinase and inhibits the progression of atherosclerotic plaque^30^. Because inflammation is an important component of MetS^31^ and vitamin D is anti-inflammatory, MetS might be linked to vitamin D deficiency. We chose hsCRP as the inflammation biomarker because it predicts cardiovascular risk^32^. Observational studies^33–37^ report inconsistent and variable results for the association of inflammatory markers and serum 25(OH)D. One study^38^ reported that bi-directional Mendelian randomization analyses showed no evidence of a causal relationship between high levels of 25(OH)D and low levels of CRP. One large observational study^39^ reported, in an asymptomatic general population, a significant inverse relation between 25(OH)D and serum CRP, independent of traditional cardiovascular risk factors, but only at concentrations <21 ng/mL; at concentrations ≥21 ng/mL, it was associated with an increase in serum CRP. The relatively higher 25(OH)D concentrations in our patients might have led to a nonsignificant association between 25(OH)D and hsCRP in our elderly participants. The relationship among 25(OH)D, CRP, and other inflammatory markers require additional investigations.

Although the association was not observed in our study, vitamin D deficiency was reported^40,41^ to be associated with high blood pressure, possibly through lack of suppression of the renin-angiotensin system^42,43^. The roles of vitamin D in atherogenic dyslipidemia has received less attention than has the mechanism discussed in the previous paragraph. In our study, the prevalence of hypertriglyceridemia and HDLC levels based on MetS criteria) were significantly lower in those with 25(OH)D concentrations ≥40 ng/mL. Most observational studies show that serum 25(OH)D is positively related to serum HDL-C and negatively related to total cholesterol (TC)/HDL-C and LDL-C/HDL-C ratios and TG^44^. In an animal study^45^, vitamin D receptor knockout mice expended more energy and consumed more oxygen than did wild-type mice; thus, they had less body fat and lower plasma TG and TC levels. In a recent study^46^, calcitriol suppressed hepatic triglyceride formation and reduced hepatic fat accumulation at above-physiological serum concentrations. More investigation is needed to clarify the role of 25(OH)D in lipid metabolism.

Osteocalcin is a novel IR biomarker of insulin resistance and believed to link bone and glucose metabolism. Serum osteocalcin levels are lower in middle-aged to elderly people with DM^47^ and elderly people with MetS^48^. We found that the total osteocalcin level was negatively associated with the components of the HOMA-IR index, MetS, and BMI. However, after HOMA-IR index had been adjusted for, osteocalcin was not a significant factor of MetS. Another study^49^ reported that weight loss and regular exercise significantly upregulated circulating osteocalcin. Because losing weight and exercise are important interventions against MetS and IR, osteocalcin might be a biomarker for MetS mediated by IR or obesity. Additional study is warranted.

The cutoff value of optimal serum 25(OH)D remains controversial. It is generally agreed that bone metabolism will be compromised once serum 25(OH)D falls <20 ng/mL^11^; optimal intestinal calcium absorption^50^ and suppression of serum iPTH occurs when serum 25(OH)D reaches about 30 ng/mL^51^. However, decades of debate, there is no consensus on the optimal level of 25(OH)D for predicting and preventing MetS, CVD, and other related chronic diseases. Nevertheless, it has been confirmed that patients with a lower, or even relatively adequate, 25(OH)D concentration have a higher risk of MetS. On the other hand, along with the increment of serum 25(OH)D concentration, the OR for metabolic syndrome shows a decreasing trend without an obvious ceiling effect (Fig. 5) as the highest 25(OH)D concentration in our study subjects is 70 ng/mL. Further investigation was needed to clarify the protective effect of high 25(OH)D concentration on metabolic risk.

In this study, serum 25(OH)D was an independent factor for MetS in the elderly without vitamin D deficiency. Interestingly, several meta-analyses^7,24,52–54^ have reported a limited effect of vitamin D supplementation on glucose homeostasis, diabetes prevention, adipokine levels, blood pressure control, and systemic inflammation. Studies^7,55^ have reported that the gene polymorphism of vitamin D synthesis (CYP1alpha), vitamin D transporter (DBP gene), and vitamin D receptor (ApaI, TaqI, BsmI, FokI) was associated with glucose intolerance and IR, and that it may influences the optimal effect of vitamin D supplements on preventing diabetes, IR, and glucose homeostasis. Or, perhaps vitamin D is a surrogate, like hsCRP in cardiovascular disease^32^, and is a biomarker for the integrated effect of metabolic abnormality which is not efficacious without the synchronized intervention of life style modification, exercise, weight control, etc. More studies of the beneficial effects of vitamin D supplementation on MetS risks are is warranted, especially in the elderly without a severe vitamin D deficiency.

## Limitations

Our study has some limitations. First, this is a cross-sectional study, which cannot disclose causal relationships but only correlations. The actual seasonal variation of serum 25(OH)D was not determined; those variations might be trivial in Taiwanese^18–20^. Second, our participants were ambulatory and relatively healthy, but we were unable to recruit and enroll critically ill or disabled elderly. Therefore, our findings might not be applicable to people who are not ambulatory or who are bedridden. Our participants might have been relatively healthy elderly to whom the primary prevention of disease was especially important. Finally, our participants elderly Taiwanese living in a rural village, so that extrapolating our findings to the general population or to other ethnicities has to be made with caution.

## Conclusion

In the elderly without vitamin D deficiency or insufficiency, serum 25(OH)D concentrations are inversely associated with metabolic syndrome risk.

## Methods

### Study participants

Tianliao District, located in Kaohsiung City in southern Taiwan, is a tropical, agricultural community at about 22°50′N latitude, like Hawaii (18° 55′N to 28° 27′N), and about 7° farther south than Florida (24°27′N to 31°00′N). The mean annual temperature is 25.1 °C (range: 19.3–29.2 °C) and mean amount of sunshine is 212.2 hours per month (longest: 221.4 hours in July; shortest: 161.8 hours in December). In the 2012 census report, the total population of Tianliao District was 7800, and 1966 (25.2%) participants were ≥65 years old. A cross-sectional survey^56,57^ targeted Tianliao’s elderly (≥65 years old) was conducted in July 2012. After we had excluded empty houses (n = 489) and deceased (n = 40), non-ambulatory (significantly disabled: n = 138), and unreachable residents (n = 201), we finally enrolled 285 men and 264 women using the whole-district sampling method. The response rate was 50% (549/1098).

### Ethical approval

This study was approved by the Institutional Review Board of National Cheng Kung University Hospital in compliance with the World Medical Association Declaration of Helsinki and good practice (GCP) guideline. Each participant signed an informed consent before the study began.

### Data Collection: Questionnaire

Well-trained staff used a 20-minute structured questionnaire^56,57^ for individual face-to-face interviews with participants. The questionnaires asked about (a) sociodemographic characteristics: sex, age, occupational status, living status, and education; (b) lifestyle: cigarette smoking and alcohol drinking; (c) medical history: DM, hypertension, dyslipidemia, and medication; and (d) noninvasive assessment tools: the short-form International Physical Activity Questionnaires (IPAQ)^58^, the Mini Nutritional Assessment (MNA)^59^, and the 10-item Short Portable Mental Status Questionnaire (SPMSQ)^60^.

Cigarette smoking was quantified by packs/year (PYs), and habitual alcohol drinking was defined as “drinking alcohol more than once a week for more than half a year”. Physical activity was categorized by tertiles (low, middle, and high) levels)^61^. Nutritional status was categorized as normal (score: ≥24), at risk of malnutrition (score: ≥17 to <24), and malnutrition (score: <17) using the MNA^62^. Cognitive function was categorized as unimpaired to mildly impaired (score: 0–5) and moderately to severely impaired (score: 6–10)^63^.

### Anthropometric variables

Body weight (BW) to the nearest 0.1 kg, and body height (BH) to the nearest mm (Solo^®^ Eye-Level Clinical Scale; Detecto, Webb City, MO, USA) were measured with the participants wearing light clothing without shoes. The body mass index (BMI) kg/BH[m^2^]) was calculated. With the participant standing naturally, looking forward, and wearing only underwear, their waist circumference (WC) was measured to the nearest mm, using a standard tape (Gulick II; Country Technology, Inc., Gays Mills, WI, USA), midway between the lateral lower rib margin and the superior anterior iliac crest after a gentle breath expiration. Blood pressure (BP) was the average of two right-arm readings (HEM-7230; Omron, Tokyo, Japan) with the participant sitting down. Each variable was measured by the same trained staff.

### Biochemistry variables

The laboratory data (fasting blood glucose, high-density lipoprotein cholesterol (HDL-C), triglyceride, fasting blood insulin, high sensitivity C-reactive protein (hsCRP), intact parathyroid hormone (iPTH), and osteocalcin) were obtained from blood samples after participants had fasted overnight. The homeostasis model assessment insulin resistance (HOMA-IR) was obtained analyzing fasting blood glucose and fasting blood insulin^64^. Serum 25(OH)D concentrations and insulin and osteocalcin levels were measured using competitive radioimmunoassay kits (Cobas^®^; Roche Vitamin D Total first generation assay (25OHD-I), Roche Diagnostics, Indianapolis, IN, USA). The coefficients of variation were 25(OH)D: 3.1%, insulin: 1.9%, and osteocalcin: 3.3%.

### Diagnosis of MetS and vitamin D deficiency

MetS was diagnosed if a participant had three or more of the following five criteria defined by the Modified National Cholesterol Education Program Adult Treatment Panel III (ATPIII) Guideline for the Chinese population^65^: (a) fasting plasma glucose level ≥100 mg/dL or taking a hypoglycemic agent; (b) systolic blood pressure ≥130 mmHg, or diastolic blood pressure ≥85 mmHg, or taking an anti-hypertension medication; (c) serum HDL-C level <40 mg/dL (men) or <50 mg/dL (women); (d) serum triglyceride level ≥150 mg/dL; (e) WC ≥90 cm (men) or ≥80 cm (women). Although the cutoff of vitamin D deficiency and insufficiency is disputed because its high prevalence is disproportionate to actual illness, vitamin D deficiency is universally defined as a serum 25(OH)D concentration <20 ng/mL (50 nmol/L); vitamin D insufficiency is defined as a 25(OH)D concentration <30 ng/mL^11^.

### Data analysis and statistical methods

All continuous variables (age, BMI, WC, and laboratory data) are expressed as means ± standard deviation (SD). Dichotomous data (sociodemographics, habitual alcohol drinking, PYs of cigarette smoking, physical activity, nutrition status, and cognitive function) are expressed as percentages. The log transformation method for HOMA-IR and osteocalcin was used to fit them to the normal distribution model. The differences in continuous and dichotomized variables between MetS^−^ and MetS^+^ participants were analyzed using independent *t* tests or χ^2^ tests, respectively.

Pearson correlations of 25(OH)D concentration, log (osteocalcin), log (HOMA-IR), hsCRP, and BMI were calculated. The P_trend_ of 25(OH)D concentration, osteocalcin, HOMA-IR and Hba1c with components of MetS were analyzed by using ANOVA. Binary logistic regression analysis was used to assess the independent contribution to the MetS by possible associated factors: age, sex, BMI, living and literacy statuses, current employment, habitual alcohol drinking, PYs of cigarette smoking, physical activity, nutrition status, cognitive function, 25(OH)D concentration (either as a continuous or as a categorical variable), log (osteocalcin), log (HOMA-IR), and hsCRP. The Nagelkerke pseudo R^2^ test was used to describe how well the model predicted MetS. SPSS 17 for Windows was used for all analyses. Significance was set at P < 0.05 (two-tailed).

## Electronic supplementary material

Supplementary Information
