# Supplementary material for: Inverse Relationship between Metabolic Syndrome and 25-Hydroxyvitamin D Concentration in Elderly People without Vitamin D deficiency
Source: Sci Rep. 2018 Nov 19;8:17052. doi: 10.1038/s41598-018-35229-2 (PMC6242887; doi:10.1038/s41598-018-35229-2)
Supplement: Supplementary file 1 — Supplementary Information [file 41598_2018_35229_MOESM1_ESM.doc]

**Inverse Relationship between Metabolic Syndrome and 25-Hydroxyvitamin D Concentration in Elderly People without Vitamin D deficiency**

Chun-Min Wang^1^, Chin-Sung Chang^2^, Yin-Fan Chang^2^, Shin-Jiuan Wu^3^, Ching-Ju Chiu^4^, Meng-Tzu Hou^5^, Chuan-Yu Chen^2^, Ping-Yen Liu^6^, Chih-Hsing Wu^2,3*^

^1^Department of Neurology, National Cheng Kung University Hospital and College of Medicine, National Cheng Kung University, Tainan, Taiwan;

^2^Department of Family Medicine, National Cheng Kung University Hospital and College of Medicine, National Cheng Kung University, Tainan, Taiwan;

^3^Department of Food Nutrition, Chung Hwa University Medical Technology, Tainan, Taiwan;

^4^Institute of Gerontology, College of Medicine, National Cheng Kung University, Tainan, Taiwan;

^5^Department of Physical Therapy and Assistive Technology, National Yang-Ming University, Taipei, Taiwan;

^6^Department of Internal Medicine, Division of Cardiology, National Cheng Kung University Hospital and College of Medicine, National Cheng Kung University, Tainan, Taiwan

**Running head:** Metabolic Syndrome and 25-Hydroxyvitamin D Concentration in Elderly

**Corresponding author:** Chih-Hsing Wu, MD

Department of Family Medicine, National Cheng Kung University Hospital

138 Sheng-Li Road, Tainan 704, Taiwan

**Email:** paulo@mail.ncku.edu.tw; **Tel:** +886-6-235-3535, ext. 5200; **FAX:** +886-6-275-4243

**Word count:** Abstract: 204, Context: 3271, Figures: 5, Tables: 3, Appendices: 6.

**ClinicalTrials.gov Identifier:** NCT03344718

**Appendix 1.** Average serum 25(OH)D concentration of 523 elderly with and without vitamin D supplement intake*

| **Vitamin D supplement** | **n** | **Mean (ng/mL)** | **Standard Deviation** |
| --- | --- | --- | --- |
| **Yes** | 5 | 40.1 | 11.7 |
| **No** | 518 | 43.9 | 11.2 |
| **Total** | 523 | 44.0 | 11.1 |

***** independent-sample *t* tests**, p=0.56**

**Appendix 2. Demographic and laboratory data for the elderly with 20 ≤ 25(OH)D < 30 and ≥ 30** **ng/mL**

| **Variable** | **All** | **20 ≤ 25(OH)D < 30**  ng/mL | **25(OH)D ≥ 30**  ng/mL | **p value** |
| --- | --- | --- | --- | --- |
| **Number** | 523 | 49 | 474 |  |
| **Age (years)** | 76 ± 6.2 | 74.6 ± 6.0 | 76.1 ± 6.3 |  |
| **Male** | 269 (51.4%) | 11 (22.4%) | 258 (54.4%) | < 0.001 |
| **Had occupation** | 230 (44.1%) | 21 (42.9%) | 209 (44.2%) | 0.881 |
| **Lived with partner** | 436 (83.4%) | 47 (95.9%) | 389 (82.1%) | 0.009 |
| **Literate** | 268 (51.0%) | 23 (46.9%) | 245 (51.7%) | 0.551 |
| **Alcohol drinking** | 84 (16.1%) | 4 (8.2%) | 80 (16.9%) | 0.151 |
| **Smoking** |  |  |  | 0.013 |
| PY = 0 | 388 (74.5%) | 45 (91.8%) | 343 (72.7%) |  |
| 0-30 PY | 44 (8.4%) | 2 (4.1%) | 42 (8.9%) |  |
| ≥ 30 PY | 89 (17.1%) | 2 (4.1%) | 87 (18.4%) |  |
| **Physical activity (IPAQ-short form)** |  |  |  | 0.133 |
| Low | 172 (33.0%) | 15 (30.6%) | 157 (33.2%) |  |
| Middle | 171 (32.8%) | 22 (44.9%) | 149 (31.5%) |  |
| High | 179 (34.3%) | 12 (24.5%) | 167 (35.3%) |  |
| **Body mass index (kg/m^2^)** | 24.5 ± 3.8 | 25.2 ± 3.3 | 24.4 ± 3.8 | 0.188 |
| **Waist circumference (cm)** | 87.2 ± 10.1 | 86.8 ± 7.8 | 87.2 ± 10.3 | 0.783 |
| **Components of metabolic syndrome** |  |  |  |  |
| Central obesity | 319 (61.0%) | 36 (73.5%) | 283(59.7%) | 0.060 |
| Hypertension | 391 (74.8%) | 37 (75.5%) | 354 (74.7%) | 0.899 |
| High fasting glucose level | 228 (43.6%) | 28 (57.1%) | 200 (42.2%) | 0.045 |
| Hypertriglyceridemia | 127 (24.3%) | 15 (30.6%) | 112 (23.6%) | 0.278 |
| Low HDLC level | 186 (35.7%) | 19 (38.8%) | 167 (35.2%) | 0.622 |
| **Mini-nutritional assessment** |  |  |  | 0.838 |
| Malnourished (< 17.0) | 3 (0.6%) | 0 (0%) | 3 (0.6%) |  |
| At risk of malnutrition (17-24) | 90 (17.2%) | 9 (18.4%) | 81 (17.1%) |  |
| Well-nourished (≥ 24.0) | 430 (82.2%) | 40 (81.6%) | 390 (82.3%) |  |
| **SPMSQ** |  |  |  | 0.026 |
| No or mild cognitive impairment | 468 (89.5%) | 39 (79.6%) | 429 (90.5%) |  |
| Moderate to severe cognitive impairment | 55 (10.5%) | 10 (20.4%) | 45 (9.5%) |  |
| **hsCRP > 3 (mg/L)** | 103 (19.7%) | 7 (14.3%) | 96 (20.3%) | 0.450 |
| **Log (HOMA-IR)** | 0.23 ± 0.34 | 0.35 ± 0.33 | 0.11 ± 0.34 | 0.012 |
| **Log (Osteocalcin) (ng/dL)** | 1.29 ± 0.21 | 1.38 ± 0.23 | 1.28 ± 0.20 | 0.001 |

Data expressed as number (percent) or mean ± standard deviation. Continuous data were analyzed using independent-sample *t* tests; dichotomous data were analyzed using χ^2^ tests. PY: pack-year; HDLC: high-density lipoprotein cholesterol; HOMA-IR: homeostatic model assessment insulin resistance index; hsCRP: high-sensitivity C-reactive protein; IPAQ: International Physical Activity Questionnaire; SPMSQ: Short Portable Mental Status Questionnaire.

**Appendix 3. Demographic and laboratory data for the elderly with 20 ≤ 25(OH)D < 40 and ≥ 40** ng/mL

| **Variable** | **All** | **20 ≤ 25(OH)D < 40** ng/mL | **25(OH)D ≥ 40** ng/mL | **p value** |
| --- | --- | --- | --- | --- |
| **Number** | 523 | 204 | 319 |  |
| **Age (years)** | 76 ± 6.2 | 75.3 ± 5.8 | 76.4 ± 6.5 | 0.041 |
| **Male** | 269 (51.4%) | 62 (30.4%) | 207 (64.9%) | < 0.001 |
| **Had occupation** | 230 (44.1%) | 91 (44.6%) | 139 (43.7%) | 0.840 |
| **Lived with partner** | 436 (83.4%) | 171 (83.8%) | 389 (83.1%) | 0.822 |
| **Literate** | 268 (51.2%) | 88 (43.1%) | 180 (56.4%) | 0.003 |
| **Habitual alcohol drinking** | 84 (16.1%) | 15 (7.4%) | 69 (21.6%) | <0.001 |
| **Smoking** |  |  |  | <0.001 |
| PY = 0 | 388 (74.5%) | 171 (83.8%) | 217 (68.5%) |  |
| 0-30 PY | 44 (8.4%) | 8 (3.9%) | 36 (11.4%) |  |
| ≥ 30 PY | 89 (17.1%) | 25 (12.3%) | 64 (20.2%) |  |
| **Physical activity (IPAQ-short form)** |  |  |  | 0.126 |
| Low | 172 (33.0%) | 65 (31.9%) | 107 (33.6%) |  |
| Middle | 171 (32.8%) | 77 (37.7%) | 94 (29.6%) |  |
| High | 179 (34.3%) | 62 (30.4%) | 117 (36.8%) |  |
| **Body mass index (kg/m^2^)** | 24.5 ± 3.8 | 24.8 ± 3.7 | 24.3 ± 3.8 | 0.151 |
| **Waist circumference (cm)** | 87.2 ± 10.1 | 87.2 ± 9.3 | 87.2 ± 10.6 | 0.978 |
| **Components of metabolic syndrome** |  |  |  |  |
| Central obesity | 319 (61.0%) | 139 (68.1%) | 180(56.4%) | 0.007 |
| Hypertension | 391 (74.8%) | 154 (75.5%) | 237 (74.3%) | 0.837 |
| High fasting glucose level | 228 (43.6%) | 96 (47.1%) | 132 (41.4%) | 0.201 |
| Hypertriglyceridemia | 127 (24.3%) | 63 (30.9%) | 64 (20.1%) | 0.005 |
| Low HDLC level | 186 (35.7%) | 85 (41.7%) | 101 (31.7%) | 0.020 |
| **Mini-nutritional assessment** |  |  |  | 0.082 |
| Malnourished (< 17.0) | 3 (0.6%) | 3 (1.5%) | 0 (0%) |  |
| At risk of malnutrition (17-24) | 90 (17.2%) | 37 (18.1%) | 53 (16.6%) |  |
| Well-nourished (≥ 24.0) | 430 (82.2%) | 164 (80.4%) | 266 (83.4%) |  |
| **SPMSQ** |  |  |  | 0.105 |
| No or mild cognitive impairment | 468 (89.5%) | 177 (86.8%) | 291 (91.2%) |  |
| Moderate to severe cognitive impairment | 55 (10.5%) | 27 (13.2%) | 28 (8.8%) |  |
| **hsCRP > 3 (mg/L)** | 103 (19.7%) | 34 (16.7%) | 69 (21.6%) | 0.164 |
| **Log (HOMA-IR)** | 0.23 ± 0.34 | 0.28 ± 0.32 | 0.20 ± 0.35 | 0.011 |
| **Log (Osteocalcin) (ng/dL)** | 1.29 ± 0.21 | 1.30 ± 0.21 | 1.28 ± 0.21 | 0.228 |

Data expressed as number (percent) or mean ± standard deviation. Continuous data were analyzed using independent-sample *t* tests; dichotomous data were analyzed using χ^2^ tests. PY: pack-year; HDLC: high-density lipoprotein cholesterol; HOMA-IR: homeostatic model assessment insulin resistance index; hsCRP: high-sensitivity C-reactive protein; IPAQ: International Physical Activity Questionnaire; SPMSQ: Short Portable Mental Status Questionnaire.

**Appendix 4. Binary logistic regression model for factors significantly associated with the presence of metabolic syndrome in 474 elderly with serum 25(OH)D ≥ 32 ng/mL**

|  | | **Model 1** | **Model 2** | **Model 3** | **Model 4** |
| --- | --- | --- | --- | --- | --- |
| **Nagekerker R^2^** | | **0.16** | **0.39** | **0.36** | **0.45** |
| **Variable** | **Category** | **OR (95% CI)** | **OR (95% CI)** | **OR (95% CI)** | **OR (95% CI)** |
| **Age (years)** |  | 0.94 (0.91-0.98)** | 0.98 (0.94-1.02) | 0.98 (0.94-1.02) | 0.99 (0.95-1.03) |
| **Gender** | Female | 2.96 (1.61-5.43)*** | 2.5 (1.28-4.90)** | 2.78 (1.42-5.45)** | 2.44 (1.21-4.90)* |
| **BMI (kg/m^2^)** |  | － | － | 1.38 (1.27-1.49)*** | 1.25 (1.14-1.36)*** |
| **Live alone** | Yes | 1.50 (0.87-2.59) | 1.55 (0.85-2.83) | 1.40 (0.77-2.52) | 1.46 (0.78-2.73) |
| **Employed** | Yes | 0.96 (0.60-1.55) | 0.61 (0.35-1.06) | 0.83 (0.48-1.41) | 0.62 (0.35-1.11) |
| **Literate** | Yes | 0.90 (0.54-1.47) | 0.69 (0.40-1.20) | 0.82 (0.47-1.41) | 0.69 (0.39-1.24) |
| **Smoking (PY)** | PY = 0 (reference) | 1 | 1 | 1 | 1 |
|  | 0-30 PY | 1.50 (0.67-3.34) | 1.13 (0.45-2.79) | 1.81 (0.74-4.41) | 1.35 (0.52-3.48) |
|  | ≥ 30 PY | 1.51 (0.79-2.88) | 1.25 (0.59-2.64) | 1.61 (0.79-3.27) | 1.32 (0.61-2.84) |
| **Alcohol drinking** | Yes | 1.27 (0.67-2.40) | 1.72 (0.82-3.61) | 1.28 (0.63-2.57) | 1.60 (0.75-3.41) |
| **Physical activity** | Low (reference) | 1 | 1 | 1 | 1 |
|  | Middle | 0.55 (0.32-0.94)* | 0.48 (0.26-0.88)* | 0.54 (0.30-0.99)* | 0.48 (0.25-0.92)* |
|  | High | 0.59 (0.33-1.05) | 0.40 (0.20-0.78)** | 0.46 (0.24-0.90)* | 0.36 (0.17-0.73)** |
| **Malnutrition** | No (reference) | 1 | 1 | 1 | 1 |
|  | At risk | 3.25 (0.27-39.4) | 5.73 (0.17-192.7) | 1.27 (0.10-15.63) | 2.37 (0.11-51.9) |
|  | Malnutrition | 3.67 (0.31-44.2) | 6.16 (0.19-201.7) | 0.81 (0.07-9.70) | 1.77 (0.08-38.0) |
| **Mental impairment** | Moderate to severe | 1.07 (0.52-2.21) | 1.65 (0.72-3.80) | 1.20 (0.53-2.68) | 1.57 (0.66-3.71) |
| **25(OH)D (ng/mL)** |  | 0.98 (0.95-0.999)* | 0.98 (0.95-1.02) | 0.98 (0.95-1.01) | 0.98 (0.95-1.01) |
| **Log (HOMA-IR)** |  | － | 50.0 (19.6-128.3)*** | － | 18.68 (6.97-50.09)*** |
| **Log (Osteocalcin) (ng/dL)** | | 0.18 (0.06-0.53)** | 0.295 (0.09-0.95)* | 0.27 (0.07-0.88)* | 0.38 (0.12-1.28) |
| **hsCRP (mg/L)** |  | 1.02 (0.98-1.06) | 1.00 (0.97-1.04) | 1.00 (0.96-1.04) | 1.00 (0.96-1.04) |

OR: odds ratio; CI: confidence interval; PY: packs per year; HOMA-IR: homeostatic model assessment insulin resistance index; BMI: body mass index; hsCRP: high sensitivity C-reactive protein.

*p < 0.05, **p < 0.01, ***p < 0.001.

**Appendix 5. Binary logistic regression model for factors significantly associated with the presence of metabolic syndrome in 319 elderly with serum 25(OH)D ≥ 40 ng/mL**

|  | | **Model 1** | **Model 2** | **Model 3** | **Model 4** |
| --- | --- | --- | --- | --- | --- |
| **Nagekerker R^2^** | | **0.21** | **0.42** | **0.36** | **0.44** |
| **Variable** | **Category** | **OR (95% CI)** | **OR (95% CI)** | **OR (95% CI)** | **OR (95% CI)** |
| **Age (years)** |  | 0.95 (0.91-0.99)* | 0.99 (0.94-1.04) | 0.97 (0.94-1.03) | 1.00 (0.95-1.05) |
| **Gender** | Female | 3.30 (1.56-7.00)** | 3.05 (1.34-6.91)** | 2.93 (1.28-6.73)* | 2.83 (1.20-6.67)* |
| **BMI (kg/m^2^)** |  | － | － | 1.40 (1.27-1.55)*** | 1.26 (1.13-1.41)*** |
| **Live alone** | Yes | 2.6 (1.34-5.36)** | 3.02 (1.40-6.51)* | 2.65 (1.25-5.59)* | 2.92 (1.32-6.46)* |
| **Employed** | Yes | 0.91 (0.50-1.64) | 0.59 (0.30-1.18) | 0.74 (0.38-1.44) | 0.60 (0.30-1.22) |
| **Literate** | Yes | 0.80 (0.43-1.47) | 0.63 (0.32-1.25) | 0.70 (0.35-1.39) | 0.62 (0.30-1.27) |
| **Smoking (PY)** | PY = 0 (reference) | 1 | 1 | 1 | 1 |
|  | 0-30 PY | 1.41 (0.59-3.40) | 0.97 (0.36-2.63) | 1.77 (0.67-4.72) | 1.28 (0.45-3.65) |
|  | ≥ 30 PY | 1.46 (0.69-3.09) | 1.10 (0.46-2.62) | 1.54 (0.68-3.53) | 1.20 (0.49-2.94) |
| **Alcohol drinking** | Yes | 1.81 (0.10-3.66) | 2.71 (1.19-6.18)* | 1.89 (0.86-4.12) | 2.48 (1.06-5.79)* |
| **Physical activity** | Low (reference) | 1 | 1 | 1 | 1 |
|  | Middle | 0.51 (0.26-0.99)* | 0.43 (0.20-0.93)* | 0.47 (0.22-0.998)* | 0.43 (0.19-0.95)* |
|  | High | 0.67 (0.33-1.35) | 0.49 (0.22-1.10) | 0.52 (0.23-1.15) | 0.43 (0.19-1.02) |
| **Malnutrition** | At risk (reference) | 1 | 1 | 1 | 1 |
|  |  | 1.10 (0.53-2.27) | 0.84 (0.36-1.94) | 0.53 (0.22-1.27) | 0.54 (0.22-1.37) |
| **Mental impairment** | Moderate to severe | 1.26 (0.50-3.18) | 2.82 (0.91-8.74) | 1.51 (0.53-4.32) | 2.55 (0.80-8.07) |
| **25(OH)D (ng/mL)** |  | 0.95 (0.92-0.99)** | 0.95 (0.91-0.99)** | 0.95 (0.92-0.99)* | 0.95 (0.91-0.99)* |
| **Log (HOMA-IR)** |  | － | 47.6 (13.1-149.84)*** | － | 16.9 (5.10-56.14)*** |
| **Log (Osteocalcin) (ng/dL)** | | 0.27 (0.08-1.003) | 0.42 (0.10-1.71) | 0.46 (0.11-1.88) | 0.56 (0.13-2.37) |
| **hsCRP (mg/L)** |  | 1.05 (0.99-1.10) | 1.02 (0.97-1.08) | 1.01 (0.96-1.07) | 1.00 (0.95-1.06) |

OR: odds ratio; CI: confidence interval; PY: packs per year; HOMA-IR: homeostatic model assessment insulin resistance index; BMI: body mass index; hsCRP: high sensitivity C-reactive protein.

*p < 0.05, **p < 0.01, ***p < 0.001.

**Appendix 6. Binary logistic regression model for factors significantly associated with the presence of metabolic syndrome in 523 elderly without vitamin D deficiency (25(OH)D ≥ 20 ng/mL)**

|  | | **Model 1** | **Model 2** | **Model 3** | **Model 4** |
| --- | --- | --- | --- | --- | --- |
| **Nagekerker R^2^** | | **0.16** | **0.37** | **0.36** | **0.44** |
| **Variable** | **Category** | **OR (95% CI)** | **OR (95% CI)** | **OR (95% CI)** | **OR (95% CI)** |
| **Age (years)** |  | 0.95 (0.91-0.98)** | 0.98 (0.94-1.02) | 0.98 (0.94-1.02) | 0.99 (0.95-1.05) |
| **Gender** | Female | 3.04 (1.73-5.35)*** | 2.52 (1.36-4.65)** | 2.90 (1.56-5.40)** | 2.50 (1.30-4.73)** |
| **BMI (kg/m^2^)** |  | － | － | 1.37 (1.27-1.48)*** | 1.26 (1.16-1.36)*** |
| **Live alone** | Yes | 1.40 (0.84-2.34) | 1.41 (0.80-2.49) | 1.34 (0.77-2.35) | 1.37 (0.76-2.46)* |
| **Employed** | Yes | 1.13 (0.73-1.75) | 0.80 (0.49-1.32) | 0.97 (0.60-1.59) | 0.81 (0.45-1.35) |
| **Literate** | Yes | 0.96 (0.61-1.50) | 0.76 (0.46-1.25) | 0.88 (0.53-1.47) | 0.76 (0.45-1.29) |
| **Smoking (PY)** | PY = 0 (reference) | 1 | 1 | 1 | 1 |
|  | 0-30 PY | 1.55 (0.72-3.32) | 1.27 (0.55-2.95) | 1.79 (0.77-4.14) | 1.49 (0.62-3.62) |
|  | ≥ 30 PY | 1.60 (0.85-2.99) | 1.33 (0.65-2.71) | 1.57 (0.79-3.12) | 1.34 (0.64-2.81) |
| **Alcohol drinking** | Yes | 1.19 (0.65-2.17) | 1.57 (0.79-3.13) | 1.30 (0.67-2.50) | 1.56 (0.77-3.17) |
| **Physical activity** | Low (reference) | 1 | 1 | 1 | 1 |
|  | Middle | 0.58 (0.35-0.95)* | 0.52 (0.30-0.90)* | 0.60 (0.35-1.03) | 0.53 (0.30-0.95)* |
|  | High | 0.60 (0.34-1.03) | 0.47 (0.25-0.87)* | 0.50 (0.27-0.93)* | 0.43 (0.22-0.82)* |
| **Malnutrition** | No (reference) | 1 | 1 | 1 | 1 |
|  | At risk | 2.64 (0.22-32.15) | 3.95 (0.15-106.61) | 0.97 (0.08-11.96) | 1.59 (0.08-31.09) |
|  | Malnutrition | 3.08 (0.26-36.65) | 4.51 (0.17-118.82) | 0.71 (0.60-8.58) | 1.31 (0.07-25.26) |
| **Mental impairment** | Moderate to severe | 1.18 (0.62-2.25) | 1.54 (0.75-3.16) | 1.31 (0.64-2.69) | 1.51 (0.72-3.19) |
| **25(OH)D (ng/mL)** | 20 ≤ 25(OH)D < 30 (reference) | 1 | 1 | 1 | 1 |
|  | 30 ≤ 25(OH)D < 40 | 0.50 (0.24-1.02) | 0.60 (0.27-1.33) | 0.55 (0.25-1.21) | 0.60 (0.27-1.37) |
|  | 40 ≤ 25(OH)D < 50 | 0.53 (0.26-1.10) | 0.68 (0.31-1.54) | 0.59 (0.27-1.30) | 0.71 (0.31-1.60) |
|  | 50 ≤ 25(OH)D < 60 | 0.43 (0.20-0.94)* | 0.60 (0.25-1.44) | 0.48 (0.21-1.12) | 0.60 (0.25-1.44) |
|  | 25(OH)D ≥ 60 | 0.25 (0.10-0.66)** | 0.24 (0.08-0.70)** | 0.27 (0.10-0.75)* | 0.25 (0.08-0.73)* |
| **Log (HOMA-IR)** |  | － | 36.8 (16.00-84.72)*** | － | 14.59 (6.12-34.75)*** |
| **Log (Osteocalcin) (ng/dL)** | | 0.27 (0.03-0.76) | 0.27 (0.09-0.76)* | 0.23 (0.08-0.67)* | 0.32 (0.11-0.95)* |
| **hsCRP (mg/L)** |  | 1.01 (0.97-1.04) | 1.01 (0.97-1.04) | 1.01 (0.97-1.04) | 1.00 (0.96-1.04) |

OR: odds ratio; CI: confidence interval; PY: packs per year; HOMA-IR: homeostatic model assessment insulin resistance index; BMI: body mass index; hsCRP: high sensitivity C-reactive protein.

*p < 0.05, **p < 0.01, ***p < 0.001.
